# Unexpected adhesive bowel obstruction after endoscopic submucosal dissection of early sigmoid colon cancer

**DOI:** 10.1002/deo2.194

**Published:** 2022-11-30

**Authors:** Kenta Seki, Taku Sakamoto, Mai Ego Makiguchi, Naoya Toyoshima, Hiroyuki Takamaru, Masau Sekiguchi, Masayoshi Yamada, Shigeki Sekine, Yukihide Kanemitsu, Yutaka Saito

**Affiliations:** ^1^ Endoscopy Division National Cancer Center Tokyo Japan; ^2^ Gastrointestinal Endoscopy Division University of Tsukuba Hospital Ibaraki Japan; ^3^ Department of Diagnostic Pathology National Cancer Center Tokyo Japan; ^4^ Department of Colorectal Surgery National Cancer Center Tokyo Japan

**Keywords:** adhesive bowel obstruction, endoscopic submucosal dissection, micro‐perforation, post‐ESD coagulation syndrome, sigmoid colon

## Abstract

Various complications of colorectal endoscopic submucosal dissection (ESD) have been reported, including bleeding, penetration, perforation, and coagulation syndrome. However, the occurrence of bowel obstruction after ESD is rare. We report a case of adhesive bowel obstruction after ESD for a laterally spreading tumor in the sigmoid colon. The 35‐mm tumor was successfully removed by ESD without intraoperative complications. The patient had a fever, lower abdominal pain, and a small amount of bloody stool the day after ESD. Endoscopy revealed minor bleeding from the ESD scar, which was treated by hemostatic clips. Pathological analysis showed adenocarcinoma was exposed to the vertical margin; therefore, the resection was non‐curative. At 39 days after ESD and 36 days after discharge, the patient had abdominal pain and nausea. She was readmitted with a diagnosis of adhesive bowel obstruction. Conservative treatment was ineffective; therefore, she underwent sigmoidectomy combined with partial resection of the small intestine because of small intestinal stenosis caused by inflammation. The pathological examination showed localized peritonitis around the sigmoid colon where ESD was performed. There was more fibrosis along the serous surface of the small intestine than on the sigmoid colon. We concluded that there was a micro‐perforation that could not be detected by endoscopy or physical examination. This case indicates that adhesive bowel obstruction may occur as a complication of ESD.

## INTRODUCTION

Endoscopic submucosal dissection (ESD) is an endoscopic resection technique for superficial colorectal tumors that allows en bloc resection regardless of the lesion size.^1^ Moreover, complete resection of T1 colorectal cancer is possible because of the direct visualization of the submucosa and muscularis propria. En bloc resection allows a more accurate pathological evaluation of the resection margins and reduces recurrence rates compared to piecemeal endoscopic mucosal resection.^2^

However, colorectal ESD is technically difficult compared to gastroesophageal ESD because of the colorectal folds, the thin muscle layer, and operability; therefore, accidental injuries are clinically emphasized.

Bleeding, penetration, perforation, and post‐ESD coagulation syndromes (PECS) are major complications of colorectal ESD. However, the occurrence of bowel obstruction after ESD is rare. Our literature research yielded a case report of bowel obstruction thought to be caused by ESD‐induced inflammatory bowel edema, excessive air insufflation, and sedation.^3^ However, there have been no reports of adhesive bowel obstruction that required surgery after ESD.

Adhesive bowel obstruction occurs more frequently in patients with a history of inflammation in the abdominal cavity. Therefore, adhesive bowel obstruction as an ESD complication may occur when perforation occurs. According to Isomoto et al., perforation is more likely to occur in cases involving submucosal fibrosis or large tumor (≧31 mm).^4^

Herein, we present a rare case of adhesive bowel obstruction caused by peritonitis attributable to micro‐perforation that could not be diagnosed by endoscopic and physical findings and required surgery.

## CASE REPORT

A 67‐year‐old woman was referred to our hospital for the treatment of a lateral spreading tumor (LST) in the sigmoid colon. She had a history of a total hysterectomy for uterine fibroids 30 years ago. She had no abdominal symptoms and no abnormalities in the routine blood tests.

**FIGURE 1:**
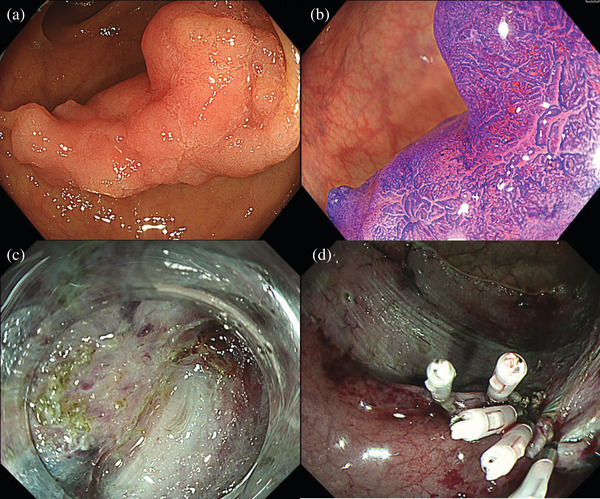
Colonoscopy findings. (a) Colonoscopy revealed a lateral spreading tumor‐nongranular measuring approximately 35 mm and of endoscopic morphological type 0–IIa+0–IIc (lateral spreading tumor‐nongranular, pseudo‐depressed type) in the sigmoid colon. (b) Magnified crystal violet staining images indicated a VI low‐grade (non‐invasive) pit pattern. (c) Intense fibrosis can be seen below the lesion. (d) Endoclips were placed on the cauterization area of the muscularis propria

Colonoscopy revealed a 35‐mm LST‐nongranular‐pseudo‐depressed type in the sigmoid colon (Figure 1a). Indigo carmine dye spraying indicated endoscopic morphological type 0–IIa+0–IIc tumor (LST‐nongranular‐pseudo‐depressed type type), and narrow‐band imaging showed Type 2B findings in the Japan Narrow‐band imaging Expert Team classification.^5^ Magnified crystal violet staining images indicated a VI slightly irregular pit pattern according to Kudo's classification, corresponding to depressed areas (Figure 1b). Therefore, we diagnosed an intramucosal to submucosal superficial carcinoma. Computed tomography showed no indications of suspected lymph node metastasis.

**FIGURE 2:**
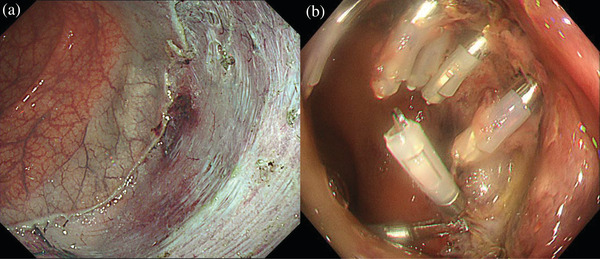
Colonoscopy findings. (a) The en bloc resection was completed without complications. (b) On day 1 after endoscopic submucosal dissection, endoclips were placed at the bleeding points of the endoscopic submucosal dissection scar

We performed ESD under intravenous anesthesia and CO_2_ insufflation. Intense fibrosis was observed just below one part of the lesion, and deep submucosal invasion of the tumor was suspected (Figure 1c). We set the cutting line just above the muscularis propria. Although there was no perforation, electrocautery damaged these parts of the muscularis propria. We placed endoclips at the locations where the burns occurred (Figure 1d) and injected ink into the contralateral wall of the ESD scar. The en bloc resection was completed in 220 min with no complications (Figure 2a). On day 1 after ESD, the patient had a fever of 37.9°C, left lower abdominal pain, and a small amount of bloody stool. Blood tests showed an increase in inflammatory response with a white blood cell count of 17,500 /μl and c‐reactive protein of 3.71 mg/dl. An emergency colonoscopy revealed bleeding from the ESD scar and no perforations were detected. We performed hemostasis using endoclips, and the bleeding was stopped (Figure 2b). She was discharged on day 3.

Pathological analysis showed a tubular adenocarcinoma invading the submucosal layer. Although the lateral margins were free of tumor, adenocarcinoma was exposed to the vertical margin, and the resection was non‐curative according to the Japanese Society for Cancer of the Colon and Rectum guidelines 2019 for the treatment of Colorectal Cancer.^6^ Therefore, additional surgical resection was considered.

**FIGURE 3:**
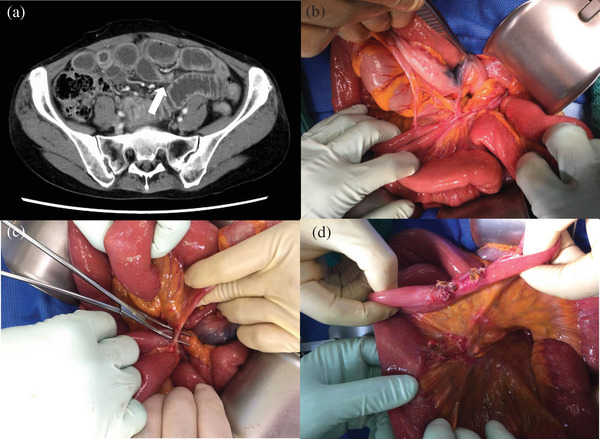
Abdominal computed tomography and operative findings. (a) The computed tomography examination revealed a caliber change in the small intestine and a dilation of the oral intestinal tract. (b, c) Serosal ink was observed in the sigmoid colon. Near the ink, the small intestine is obstructed by fibrosis and complex bands caused by partial inflammation. (d) The ileum shows stenosis caused by inflammation

She presented to our hospital 39 days after ESD because of abdominal pain and nausea. The computed tomography examination revealed a caliber change in the lower small intestine and a dilated oral intestinal lumen (Figure 3a). We inserted a decompression tube and initiated intravenous hyperalimentation. The computed tomography re‐examination revealed there was no change in the intestinal stenosis. Therefore, surgery was required. During surgery, serosal ink was detected in the sigmoid colon. Complex bands obstructing the small intestine were observed near the ink, which were coagulated and divided. However, approximately 40–50 cm from the ileocecal valve, the ileum showed stenosis caused by inflammation (Figure 3b–d). We performed sigmoidectomy (with D2 lymph node dissection) and partial resection of 12 cm of the small intestine. She was discharged 18 days after surgery without any significant complications.

**FIGURE 4:**
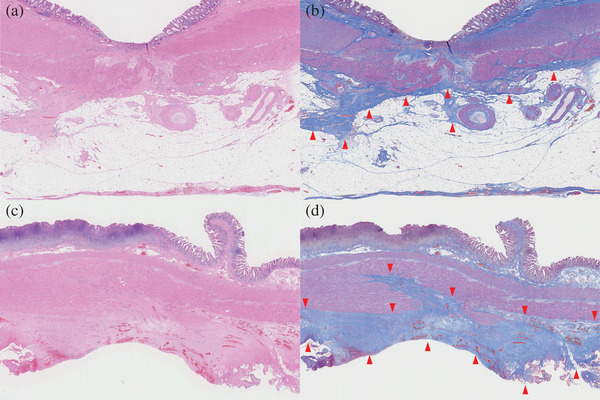
Histological findings. (a, b) Histology of the sigmoid colon. The ulcer scar with fibrosis extends to the subserosa at the endoscopic submucosal dissection site. Masson trichrome staining (blue) highlights the fibrotic areas (arrowheads). (c, d) Histology of the small intestine. Prominent fibrosis of the subserosa is observed, but the muscularis propria is intact. Masson trichrome staining (blue) highlights the fibrotic areas (arrowheads)

The pathological analysis identified no residual tumor and no lymph node metastasis. In the sigmoid colon, there was fibrosis extending to the subserosal layer at the site of ESD (Figure 4a,b). Additionally, there was extensive fibrosis and granulation tissue formation on the serosal side of the small intestine (Figure 4c,d). Based on these findings, the cause of adhesive bowel obstruction was thought to be localized peritonitis caused by ESD‐related micro‐perforation.

## DISCUSSION

In this case, the perforation was not detected by endoscopy during and after the ESD procedure but was pathologically diagnosed as adhesive bowel obstruction caused by peritonitis. A perforation during or after ESD is a severe complication of colorectal ESD because it spreads colorectal bacteria and feces into the abdominal cavity, causing peritonitis. Adhesive bowel obstruction may occur from a few months to several years after inflammation in the abdominal cavity,^7^ without any prominent inciting events. Surgery is necessary when fasting or intestinal decompression does not improve bowel obstruction. Surgery is often highly invasive because it is difficult to perform laparoscopically and requires a laparotomy.

In this case, the patient had abdominal pain, fever, and leukocytosis on 1 day after ESD, but the abdominal pain was localized and mild, and the symptoms improved the next day. Therefore, we did not suspect a perforation and diagnosed PECS. Furthermore, the colonoscopy showed no evidence of perforation. However, during surgery for adhesive bowel obstruction, adhesions were found in the vicinity of the sigmoid colon where ESD was performed. Histological findings were an ulcer scar with fibrosis extending to the subserosa at the site of ESD and prominent fibrosis surrounding the serosa of the small intestine near the ESD scar. Therefore, we considered the existence of micro‐perforation that could not be diagnosed with endoscopic and physical findings.

One possible reason for micro‐perforation is thermal damage during the cauterization of perforating arteries from the muscularis propria. When cauterizing the perforating arteries, we use bipolar hemostatic forceps to avoid thermal damage to the intestinal wall. However, we thought there was a risk of perforation because the cutting line was set just above the muscularis propria. Micro‐perforation can also be caused by endoclips placed on the muscular layer, which can result in fibrosis spreading to the subserosal layer.

Based on the definition of post‐polypectomy electrocoagulation syndrome, PECS is characterized by local abdominal pain, fever, increased inflammatory response on blood tests, and without perforation.^8^, ^9^ As most cases of PECS improve with conservative treatment, the actual condition of the abdominal cavity is unknown.

We suppose that some cases with PECS may involve micro‐perforation, and the frequency of perforation as a complication of colorectal ESD may be much higher than that reported.^10^ Therefore, in the case of abdominal pain and fever after ESD, we should not quickly diagnose PECS based on endoscopic and physical examinations.

In conclusion, adhesive bowel obstruction caused by micro‐perforation during ESD is a rare but serious complication that may require surgery. The present case indicates that adhesive bowel obstruction may occur as an ESD complication even in the absence of endoscopically detectable perforation. It is important to share case reports of ESD‐related complications to improve the technique for safer procedures and respond quickly to any complications.

## CONFLICT OF INTEREST

None.
